# Longitudinal biomarkers in dementia with Lewy bodies: A systematic review and meta-analysis

**DOI:** 10.1016/j.prdoa.2026.100470

**Published:** 2026-06-17

**Authors:** Juliette L. van Alphen, Federico E. Pozzi, Jan Booij, Frederik Barkhof, Mara ten Kate, Charlotte E. Teunissen, Wiesje M. van der Flier, Afina W. Lemstra

**Affiliations:** a Alzheimer Center Amsterdam, Neurology, Vrije Universiteit Amsterdam, Amsterdam UMC location VUmc, PO Box 7057, Amsterdam, the Netherlands; b Amsterdam Neuroscience, Neurodegeneration, ADORE Building, PO Box 7057, Amsterdam, the Netherlands; c Neurology Department, Fondazione IRCCS San Gerardo dei Tintori, Via Giovanni Battista Pergolesi 33, 20900 Monza, Italy; d Department of Radiology & Nuclear Medicine, Amsterdam UMC, University of Amsterdam, PO Box 7057, Amsterdam, the Netherlands; e Queen Square Institute of Neurology and Centre for Medical Image Computing, University College London, WC1E 6BT London, UK; f Neurochemistry Laboratory, Department of Laboratory Medicine, Amsterdam Neuroscience, Neurodegeneration, Amsterdam UMC, VU University Amsterdam, PO Box 7057, the Netherlands; g Department of Epidemiology and Data Sciences, Vrije Universiteit Amsterdam, Amsterdam UMC, PO Box 22660, Amsterdam, the Netherlands; h Alzheimer Nederland, Amersfoort, the Netherlands; i Amsterdam Public Health, Amsterdam, the Netherlands

**Keywords:** Systematic review, meta-analysis, Dementia with Lewy bodies, Longitudinal biomarkers, Disease progression

## Abstract

**Introduction:**

Dementia with Lewy bodies (DLB) is the second most prevalent neurodegenerative dementia. It is clinically and biologically heterogeneous, yet longitudinal biomarker studies assessing different aspects of neuropathological processes in DLB are scarce. We systematically reviewed the literature to identify biomarkers suitable for tracking disease progression in DLB and to evaluate potential outcome measures for clinical trials by examining which biomarkers show longitudinal changes correlating with clinical progression.

**Methods:**

Following PRISMA guidelines, we searched CENTRAL, MEDLINE, Embase, Scopus, and Web of Science for longitudinal biomarker studies in DLB (sample size ≥10, follow-up duration ≥6 months). We conducted meta-analysis of three structural MRI-studies assessing whole-brain atrophy.

**Results:**

Of 9162 titles screened, 17 studies met selection criteria including ±460 patients with MCI-LB/DLB. 15 studies included imaging biomarkers (MRI, SPECT, PET) and two studies included fluid biomarkers (plasma, CSF). Except for DAT-SPECT, most studies addressed non-DLB specific neurodegeneration or Alzheimer's disease (AD) pathology. Structural MRI-studies showed greater longitudinal atrophy in AD and mixed DLB + AD versus DLB and controls. Meta-analysis confirmed that DLB-patients were comparable to controls (*p* = 0.37), but showed lower atrophy rates than AD (*p* = 0.01). Amyloid-PET and biofluid studies suggested that amyloid accumulation follows typical progression, even in non-AD diagnoses. DAT-SPECT and FDG-PET demonstrated longitudinal changes correlating with clinical progression, showing promise as monitoring biomarkers.

**Conclusion:**

No single biomarker currently suffices to track disease progression in DLB. DAT-SPECT and FDG-PET showed promise, but further research is warranted on these and alternative, more disease-specific, accessible and feasible modalities to improve disease monitoring in future clinical trials.

## Highlights

• Outcome measures for assessing disease progression in DLB are needed.
• Monitoring biomarkers help assess target engagement and treatment effects in trials.
• Comorbid AD pathology has a prominent role in disease progression in DLB.
• FDG-PET and DAT-SPECT show promise as monitoring biomarkers, but studies are scarce.
• This review indicates that no single biomarker is yet suitable for monitoring DLB.

## 1 Introduction

Dementia with Lewy bodies (DLB) is the second most common form of dementia after Alzheimer's disease (AD) [1]. Core clinical features include symptoms of parkinsonism, visual hallucinations, cognitive fluctuations and REM-sleep behavior disorder (RBD) [2]. The pathological hallmark of DLB is the widespread accumulation of Lewy bodies and Lewy neurites, mainly composed of insoluble aggregates of phosphorylated α-synuclein, in the central and peripheral nervous system [3]. Up to 88% of DLB patients have comorbid AD-pathology, with amyloid plaques and tau neurofibrillary tangles at autopsy [4], [5].

DLB is clinically and biologically a heterogeneous disease, which complicates efforts to draw inferences about disease trajectories. To assess disease progression and to gain a better understanding of underlying disease mechanisms, reliable outcome measures are required that detect clinical and pathological decline. These outcome measures may include biomarker-based outcomes, assessed in parallel with clinical measures.

Biomarkers directly or indirectly reflect the underlying neuropathology of the disease. According to the Biomarkers, EndpointS, and other Tools (BEST) framework proposed by the FDA and NIH [6], biomarkers can be classified based on their intended use, including monitoring, diagnostic and prognostic purposes, among others. Monitoring biomarkers are defined as biomarkers measured serially to track the state of a disease over time, and can be used to assess disease progression, providing tools needed for the assessment of treatment effects in future disease-modifying trials. This distinguishes them from diagnostic biomarkers, which aim to detect the presence of a disease, and prognostic biomarkers, which aim to identify the likelihood of a clinical outcome. In the context of DLB, biomarker modalities under investigation include structural and functional neuroimaging, neurophysiological measures and fluid biomarkers. As reflected by systematic reviews on the diagnostic accuracy of DLB biomarkers [8], [9], the existing MCI-LB and DLB literature has predominantly evaluated these modalities in a (differential) diagnostic and cross-sectional context rather than serial study designs. As such, the potential of these modalities to perform as monitoring biomarkers remains largely uncharacterized.

We therefore conducted a systematic review and meta-analysis of longitudinal biomarker studies in DLB. We aimed to identify which biomarkers are best suited to track disease progression by serially assessing the state of the disease, and to identify gaps in current knowledge to guide future research. While an existing selective review provides a broader overview of diagnostic, prognostic and emerging biomarkers in DLB [10], our assessment focused on whether biomarkers demonstrated significant longitudinal change and whether such change correlated with clinical progression.

## 2 Methods

We conducted our systematic review according to Preferred Reporting Items for Systematic Reviews and Meta-Analyses (PRISMA) guidelines [11].

### 2.1 Search strategy

**Fig. 1:**
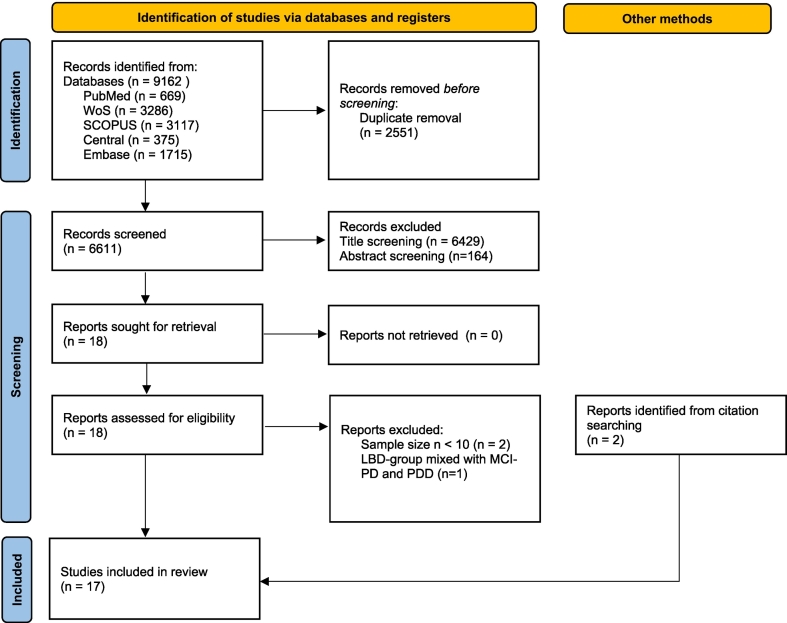
PRISMA flow-chart of study selection and inclusion.

We performed electronic searches for eligible studies up to February 1st 2025 within the databases: CENTRAL, MEDLINE, Embase, Scopus, and Web of Science (see Fig. 1). The search date did not differ between databases. Records were identified in each database with tailored search strings, that can be found in the Supplementary Material: S1. In summary, the search strings contained keywords related to the type of study (longitudinal/trajectory), type of biomarker and diagnosis.

### 2.2 Selection criteria

Inclusion criteria:

- Repeated measures of biomarkers (imaging/biofluids/neurophysiology) over time;
- Subjects include patients with diagnosis of MCI-LB and/or possible/probable DLB [2], [12], [13], [14];
- Studies on human subjects, including cohort studies (prospective or retrospective) and case series;
- Follow-up duration ≥6 months;
- Sample size ≥10.
- Full text in English language.

Exclusion criteria:

- Cross-sectional studies, case reports, conference abstracts, letters, editorials.

### 2.3 Article selection process

Citations identified from the literature searches were imported to EndNote 21. Duplicates were automatically removed by the software. Two reviewers (JA, FP) independently screened the results. In case of disagreement about eligibility, consensus was reached through discussion. Eligibility based on the selection criteria was assessed manually through: 1) title screening, 2) abstract screening, and 3) full text screening. Separate studies with the same study sample but different outcome measures were permitted. Finally, we screened reference lists of included articles to identify additional eligible studies. All decisions were checked by a third reviewer (AL).

### 2.4 Data extraction and analysis

Two reviewers (JA, FP) extracted data on: study design, sample characteristics, biomarker and clinical outcome measures, findings, strengths and limitations. Studies were categorized by biomarker type, and results were reviewed for biomarker and clinical trajectories. These results were summarized, compared and narratively described in the results section of this systematic review. Key outcomes, including effect sizes if reported, were also summarized in Table 1. Meta-analysis was conducted when ≥3 studies assessed overlapping biomarker type, cohort(s), outcome measures and reported effect size with variance. We used a random-effects model with mean difference with a fixed-effect model as sensitivity analysis to assess the robustness of the findings of the primary model. All analyses were completed in the R Studio (v4.2.1) meta package (v8.1–0).

**Table 1:** Characteristics and key outcomes of included studies.

Study	Study design			Sample(s) characteristics						Quality	Key longitudinal findings	
	Repeated biomarker	Outcome measures biomarker & clinical	Interval time for MCI-LB/DLB (in months)	Total number of longitudinal cohort	N MCI-LB/DLB^a^	Criteria for enrollment for MCI-LB/DLB	Age MCI-LB/DLB (in years)	M:F MCI-LB/DLB	N control groups	Score overall quality assessment and appraisal	Biomarker	Clinical
magnetic resonance imaging (MRI)												
O'Brien 2001 [21]	structural MRI	whole brain atrophy rate & MMSE, CDR	±12	48	10	1996	74.4 ± 6.1	8:2	9 CE, 9 VaD, 20 age-matched CU	9/10, high	Significantly lower whole brain atrophy rates in CU vs AD (*p* = 0.001) and VaD (*p* = 0.002) and a trend for DLB (*p* = 0.071). No significant differences between AD, VaD and DLB.	Associations between biomarker change and clinical change not specified.
Whitwell 2007 [26]	structural MRI	whole brain atrophy rate, ventricular expansion & MMSE, CDR	DLB 15.6 (12−30)^b^ mixed DLB + 14.4 CE (9.6–32.4)^b^	56	22: 9 DLB + 13 mixed DLB + AD	2005	DLB 72 (59–82)^b^ mixed DLB + 76 CE (52–86)^b^	12:10	12 CE, 12 FTLD-U, 5 CBD, 5 PSP, 25 age & sex-matched CU	8/10, intermediate	Significantly more whole brain atrophy and ventricular expansion in mixed DLB + AD, AD, FTLD-U, CBD and PSP vs CU (*p* < 0.01). No difference between DLB and CU for whole brain atrophy (*p* = 0.8) or ventricular expansion (*p* = 0.07). No differences among DLB, mixed DLB + AD and AD.	Associations between biomarker change and clinical change not specified.
Mak 2015⁎[23]	structural MRI	spatiotemporal patterns of cortical thinning, subcortical atrophy & CAMCOG, MMSE, UPDRS-III	12.5 ± 0.6	69	13	2005	77.0 ± 8.3	12:1	23 CE, 33 age & sex-matched CU	9/10, high	Greater % change cortical thickness (PcCTh) in AD vs DLB in left middle and superior temporal gyrus. More hippocampal atrophy in AD vs CU (*p < 0*.001) and DLB (*p* = 0.006). Steeper ventricular expansion in AD vs CU (*p* < 0.001) and DLB (*p* = 0.008). No difference in PcCTh, subcortical atrophy and ventricular expansion between DLB and CU.	Association between PcCTh in left frontal lobe and decline in MMSE, CAMCOG Orientation and Expressive Languages scores. Association between increased PcCTh in right superior parietal region and decline in UPDRS.
Mak 2015⁎[22]	structural MRI	percent whole brain volume change (PBVC), regional atrophy & CAMCOG, MMSE	12.5 ± 0.6	72	14	2005	77.2 ± 8.0	13:1	25 CE, 33 age & sex-matched CU	9/10, high	Steeper PBVC in AD vs CU (*p* < 0.01) and DLB (*p* = 0.01). No difference in PBVC in DLB vs CU (*p* = 0.95). More regional atrophy in AD vs DLB in temporal, frontal, parietal and periventricular areas (*p* < 0.05), same for CU except for occipital lobe instead of frontal lobe. No difference in regional atrophy in DLB vs CU.	No associations between PBVC and annualized change scores of MMSE and CAMCOG in combined dementia group (AD and DLB).
Nedelska 2015 [27]	structural MRI	pattern and magnitude of atrophy rates & MMSE, DRS, UPDRS-III	21.0 ± 7.0^c^	87	42 (20 DLB + 22 mixed DLB + AD)	2005	77.8 ± 8.4^cd^	16:26^c^	30 CE, 15 CU	9/10, high	Whole brain atrophy rate: higher in AD and DLB + AD vs DLB (*p* < 0.001 and *p* = 0.01, respectively). No difference between DLB and CU (*p* = 0.92). Higher in DLB + AD vs CU (*p* = 0.04), similar in DLB + AD and AD (*p =* 0.36). Similar for ventricular expansion, temporoparietal cortex, hippocampus and amygdala for, but with different effect sizes.	Associations between higher rate of atrophy in: whole brain and decreased MMSE (*p < 0*.001) and increased UPDRS (*p* = 0.0091) & hippocampus and decreased MMSE (*p* < 0.001), decreased DRS (*p* = 0.0036) and increased UPDRS (*p* < 0.001) & amygdala and decreased MMSE (*p* = 0.012), descreased DRS (*p* = 0.0022) and increased UPDRS (*p* = 0.0027). None for ventricular expansion.
Sarro 2016 [28]	structural MRI	regional atrophy in 10 ROIs, ventricular expansion & MMSE, CDR-SoB	30.0 ± 13.7	20	20	2005	70.0 ± 7.5	17:3	–	7/10, intermediate	Higher baseline amyloid-burden was associated with accelerated atrophy in the posterior cingulate gyrus (*p* = 0.023), medial temporal lobe (*p* = 0.012), occipital lobe (*p* = 0.016), temporal lobe (*p* = 0.047), caudate nucleus (*p* = 0.007) and putamen (*p* = 0.009) and ventricular expansion (*p* = 0.007).	No association between brain atrophy rate and change in MMSE-score. Association between ventricular expansion rate and increase in CDR-SoB (*p* = 0.01).
Kantarci 2022 [24]	structural MRI	regional atrophy rates & CDR-SoB	15.6 ± 10.8	168	56 (MCI-LB)	criteria for MCI^e^	70.5 ± 7.1	53:3	112 age & sex-matched CU	9/10, high	Overlap between voxel- and atlas-based analyses: more longitudinal atrophy in temporoparietal regions in MCI-LB vs CU. Atlas-based only: more atrophy in 20 ROIs in progressed MCI-LB vs CU, more atrophy in 1 ROI in stable MCI-LB vs CU. No differences in atrophy rates between progressed MCI-LB and stable MCI-LB.	Change in CDR-SoB was associated with rate of volume loss in the fusiform cortex (*p* = 0.026).
Firbank 2016⁎[29]	diffusion tensor MRI	mean diffusivity and fractional anisotropy & CAMCOG, MMSE, HVLT, BVMT, Delis Kaplan letter fluency task, UPDRS-III	12.5 ± 0.6	69	14	2005	77.2 ± 8.0	13:1	23 CE, 32 CU	9/10, high	No significant change in MD or FA. Change in MD and FA was not associated with brain atrophy.	No associations between longitudinal changes in MD or FA and cognitive measures or symptoms of parkinsonism.
Chiu 2024 [18]	diffusion weighted MRI	regional free-water change & MDS-UPDRS, MoCA	±12 and/or ± 24 months	59	39^f^: 23 with 12 m FU (20 DLB + 3 MCI-LB) and 16 with 24 m FU (14 DLB + 2 MCI-LB)	DLB 2017, MCI-LB 2020	68.6 ± 9.4^c^	38:1^c^	20 age & sex-matched CU	8/10, intermediate	Free water: Increased in 12 ROIs in DLB/MCI-LB at FU1 & FU2 + 18 ROIs at FU2 only. No longitudinal changes in CU. Longitudinal changes in 12 ROIs associated with DLB-diagnosis.	12 m FU – association between FW increase in right insula and increased MDS-UPDRS (*p* = 0.024) 24 m FU – associations between FW increase in: right amygdala, anterior SN, left fusiform, left inferior frontal operculum and increased MDS-UPDRS-total score (*p* = 0.003) & in right amygdala and increased MDS-UPDRS-III (*p* < 0.001) & in left inferior frontal operculum and decreased MoCA score (*p* = 0.004).
positron emission tomography (PET)												
Nedelska 2019 [32]	amyloid-PET ([^11^C] Pittsburgh Compound B) and structural MRI	rate of change in SUVR & MMSE, DRS, CDR-SoB, UPDRS-III, AVLT, BNT, TMT-A, RCF	14.4 ± 4.8	175	35: 33 DLB + 2 MCI-LB	2017	69.9 ± 7.3	31:4	140 age & sex-matched CU	9/10, high	No difference in rate of change in PiB SUVRs between DLB and CU. For both, the trajectory characterizes an inverted U-shaped curve over time.	Association between greater change in PiB SUVR and faster clinical and cognitive decline, measured by CDR-SoB (*p* = 0.04) and the delayed recall component of the Auditory Verbal Learning Test (*p* = 0.02).
Chen 2022 [25]	tau-PET ([^18^F]flortaucipir) and structural MRI	regional rate of change in SUVR, rate of regional atrophy & CDR-SoB, MMSE	19.2 ± 8.4	42	22	2017	70.7 ± 5.9	20:2	22 age & sex-matched CU	8/10, intermediate	Steeper rate of flortaucipir SUVR and greater rates of atrophy in DLB vs CU in multiple ROIs, but effects did not survive FDR correction. Association between increase in flortaucipir SUVR and middle temporal pole atrophy (*p* = 0.008). Additional associations between SUVR and atrophy did not survive FDR correction.	Increase in flortaucipir SUVR in the fusiform gyrus and superior and middle occipital cortices associated with decreased MMSE-score (*p* = 0.002; *p* = 0.047; *p* = 0.035). Increase in flortaucipir SUVR in the fusiform gyrus and middle occipital cortex associated with increased CDR SoB-score (*p* = 0.043; *p* = 0.014). Increase in flortaucipir SUVR in meta-ROI (fusiform gryrus + superior and middle occipital cortices) associated with decreased MMSE- (*p* = 0.014) and increased CDR-SoB score (*p* = 0.045).
Ferreira 2025 [19]	[^18^F]FDG PET	rate of change in SUVR & CDR-SoB	34.8 ± 22.8	172	72: 35 DLB + 37 MCI-LB	DLB 2017, MCI-LB 2020	69.6 ± 8.2	66:6	100 age & sex-matched CU	8/10, intermediate	Faster FDG-SUVR rate of decline in MCI-LB and DLB versus CU in: inferior temporal (*p* < 0.001), lateral frontal (*p* = 0.056; *p* < 0.001), lateral temporal (*p* < 0.001), medial temporal (*p* = 0.006; *p* = 0.003), occipital (*p* = 0.003; *p* = 0.001), parietal (*p* = 0.003; *p < 0*.001), posterior cingulate *(p < 0*.001) and temporal pole cortices (*p* = 0.009; *p < 0*.001) and meta-ROI (*p < 0*.001). Additionally in DLB only: anterior middle cingulate (*p* = 0.052), insula (*p* = 0.007) and medial frontal orbital cortices (*p < 0*.001). Pathology confirmed subset: findings did not survive FDR correction in n = 10 DLB. Faster rate of change in all ROIs (except amygdala and SN) and meta-ROI in n = 8 DLB + AD.	Significant correlations between FDG-SUVR rate of decline and CDR-SoB rate of increase in all ROIs and meta-ROI.
single photon emission computed tomography (SPECT)												
Colloby 2005 [30]	[^123^I]FP-CIT SPECT	rate of progression of nigrostriatal dopaminergic loss (measured as specific striatal binding ratios) & MMSE, CAMCOG, UPDRS-III	12.7 ± 1.2	77	20	1996	74.8 ± 7.0	14:6	20 PD, 15 PDD, 22 age-matched CU	8/10, intermediate	Reduced uptake in DLB in the anterior putamen (*p* = 0.005*),* posterior putamen (*p* = 0.02*)* and caudate nucleus (*p* = 0.06). Greater rate of decline vs CU in caudate (*p* = 0.01), posterior (*p =* 0*.*001) and anterior putamen (*p* = 0.05), no difference in rate of decline vs PD(D).	Correlation between a greater percentage of posterior putaminal dopaminergic loss and CAMCOG (*p* = 0.02).
Firbank 2005 [31]	[^99m^Tc]HMPAO SPECT	brain perfusion & CAMCOG, UPDRS-III	12.6 ± 1.1	69	18	1996	74.1 ± 7.4	10:8	17 PDD, 34 CU	8/10, intermediate	Increase in perfusion in DLB vs CU in left putamen (*p* = 0.002). Not found for PDD.	Increase in perfusion in the right caudate correlated with increase in UPDRS score (*p* = 0.021).
Durcan 2023 [16]	[^123^I]FP-CIT SPECT	rate of progression of nigrostriatal dopaminergic loss (measured as specific striatal binding ratios) & ACE-R, UPDRS	19.2 ± 10.8	85	36: 11 possible MCI-LB + 25 probable MCI-LB	2020 + criteria for MCI^g^	73 ± 7	29:7^c^	20 MCI-AD, 29 age-matched CU	8/10, intermediate	Reduced putaminal uptake in possible MCI-LB (*p* = 0.018), and probable MCI-LB (*p* = 0.008). No difference between groups in annual % change in uptake in whole striatum or striatal subregions. Steeper median annual decline in putamen vs caudate nucleus in both groups.	Correlation between decline in striatal SBR and ACE-R (r = 0.15, *p* = 0.021) and UPDRS (r = −0.14, *p* = 0.020) over time. Differences between possible/probable MCI-LB not reported.
biofluids												
Thomas 2022 [20]	plasma: p-tau181	rate of change in plasma p-tau181 concentration & ACE-R	±12–24	37	MCI-LB. Number not specified.	2020	Not specified.	Not specified.	MCI-AD (number not specified)	7/10, intermediate	3% yearly increase (*p* = 0.469). No difference in rate of change between MCI-AD and MCI-LB.	No association between 3% non-significant rate of change and cognitive decline.
Jain 2024 [17]	CSF: Aβ_42_, Aβ_40_, Aβ_42/40_, p-tau181, t-tau	change in AD-biomarker levels, ATN-status & MoCA, UPDRS-III	±12	62	27	2017	Not specified.	Not specified.	5 CE, 2 MCI-AD, 28 CU	7/10, intermediate	Increases in Aβ_40_ and Aβ_42_ (*p* < 0.0001). Increases in Aβ_42/40_-ratio, Aβ_40_ and Aβ_42_ in A + T-N- & A + T-N+ (*p* = 0.0128, *p* = 0.0007 and *p* < 0.0001, respectively) and increases in Aβ_40_ and Aβ_42_ in A + T + N- & A + T + N+ (both *p* < 0.0001). Some DLB patients classified as A + T- transitioned to A-T- over time, this was not the case for DLB with A + T+.	No significant longitudinal changes in MoCA or UPDRS-III.

⁎ = studies on approximately the same sample; DLB + AD = DLB + AD copathology; Criteria = McKeith consensus criteria (year reported), unless stated otherwise; Continuous data are presented as mean ± standard deviation unless stated otherwise; CU = cognitively unimpaired controls; Interval time for MCI-LB/DLB (in months) = time from baseline to repetition of measures.

a Diagnosis at baseline. Number of conversion at follow-up from MCI-LB to possible/probable DLB not reported. ^b^ Median (minimum-maximum). ^c^ Combined for both groups. ^d^ At second MRI. ^e^ Petersen et al. 1999 and 2009 [44][45]. ^f^ There were 23 participants that completed the first follow-up, 16 participants that completed the second follow-up and 12 participants that completed both. This suggests that there were 27 unique individuals in the study sample, however this is not further specified in the study. ^g^ Albert et al. 2011 [46].

### 2.5 Quality assessment

The methodological quality of the included studies was assessed using an adapted 11-point critical appraisal checklist for cohort studies, developed by the Johanna Brigg Institute [15]. The overall quality of the study was low if the score was ≤6, intermediate if the score was 7–8 and high if the score was ≥9.

## 3 Results

### 3.1 Selection and inclusion of studies

We identified 9162 records. After duplicate removal, 6611 titles were screened, and 6429 were excluded. During abstract screening, another 164 records were excluded. Three additional records were excluded because the DLB-sample was too small or did not solely consist of MCI-LB/DLB. By reviewing the reference lists of the 15 remaining reports, two more studies were found that met the selection criteria, adding up to a total of 17 studies included in this review (see Fig. 1).

### 3.2 Characteristics of included studies

**Fig. 2:**
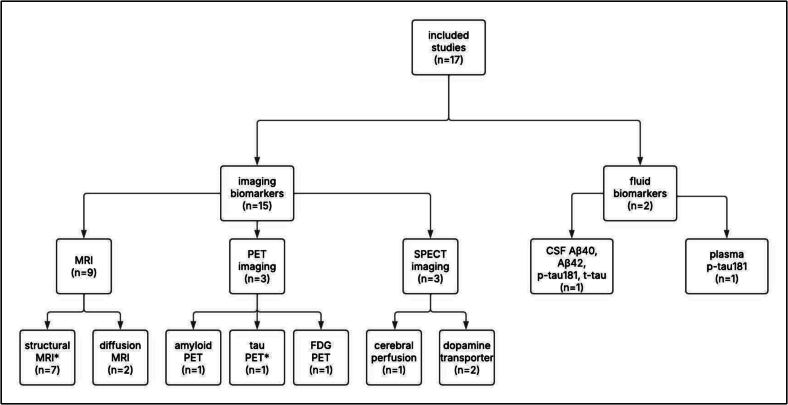
Number and specification of biomarker types of included studies.

Of the 17 studies that met the selection criteria, 15 studies reported data on imaging biomarkers and two studies reported data on fluid biomarkers. The 15 imaging studies included seven using structural MRI, two using diffusion MRI, three using SPECT (dopamine transporter and cerebral perfusion imaging), and three using PET (amyloid-PET, tau-PET and FDG-PET). The two fluid biomarker studies included one study on plasma p-tau181 and one study on CSF Aβ_42_, Aβ_40_, p-tau181 and t-tau (see Fig. 2).

In Table 1, characteristics regarding design, participant sample(s) and key findings, as well as overall quality of the included studies are presented. A complete overview of the quality assessment can be found in the Supplementary Material: S2. The most reported confounders that were corrected for in statistical models were: age, sex, duration of symptoms and follow-up time.

Two studies performed longitudinal analyses on subcohorts within originally cross-sectional study designs [16], [17], though subcohort numbers and characteristics were not specified, requiring approximations when combining data. Across all studies, ±460 with MCI-LB/DLB underwent repeated biomarker measurements. The average follow-up duration was ±18 months. Participants were predominantly male (±77%), with a mean age of ±73 years old. There was variation across studies in matching cognitively unimpaired controls to DLB for age and/or sex. All studies repeated the biomarker measurement at least once, with 4 studies assessing >2 timepoints [16], [18], [19], [20].

### 3.3 Imaging biomarkers: MRI

#### 3.3.1 Structural MRI

Out of nine MRI studies, seven investigated longitudinal brain atrophy with structural MRI. There was considerable heterogeneity in study design regarding population characteristics and methodology. This included variability in scanner types (field strengths ranging from 1 to 3 Tesla), image analysis techniques (voxel-based methods and/or region-of-interest (ROI) approaches), processing pipelines, primary outcome measures and the way results were reported.

Three MRI studies compared atrophy rates in AD, DLB and controls, without accounting for AD-copathology in DLB. Overall, DLB showed no significant longitudinal atrophy differences versus controls and lower rates than AD. O‘Brien et al. [21] found only a trend toward higher annual whole-brain atrophy rates in 10 DLB patients compared to 20 controls, whereas AD (*n* = 9) and vascular dementia (n = 9) differed significantly from controls. Differences between dementia groups were not significant, possibly due to small sample sizes. Mak et al. [22], [23] conducted two studies on similar samples: one on whole-brain atrophy and one on regional/subcortical atrophy. DLB (*n* = 14) and controls (*n* = 33) did not differ in whole-brain atrophy or regional atrophy, but both had lower atrophy than AD (*n* = 25) after ±1 year. Regionally, for DLB this was evident in temporal, frontal, parietal and periventricular areas. When focusing on subcortical structures, AD showed greater hippocampal atrophy and lateral ventricular expansion than DLB and controls, again with no significant differences between DLB and controls.

One study had post-mortem confirmed diagnoses in a subset of patients (*n* = 18/56), providing data on AD pathology. However, comorbid AD pathology was not accounted for in MRI-analyses, due to variable intervals between first MRI and death. Only MCI-patients were included at baseline, evenly divided into stable-MCI and progressed-to-dementia at follow-up. Compared with controls (*n* = 112), MCI-LB patients showed steeper atrophy rates in regions innervated by cholinergic projections of the nucleus basalis of Meynert (NBM). In stable MCI-LB, this was limited to the entorhinal parahippocampus, whereas progressed MCI-LB showed steeper atrophy in 20 ROIs, suggesting predictive value of brain atrophy in these regions for conversion to dementia. Greater baseline NBM atrophy was observed in the full MCI-LB cohort compared to controls, but atrophy rates did not differ between stable/progressed-MCI, suggesting early NBM-degeneration that plateaus and shapes subsequent neurodegeneration [24]. A longitudinal [^18^F]flortaucipir tau-PET study with repeated MRI scans classified DLB-patients as amyloid-β positive or negative using [^11^C]PiB amyloid-PET, but this was not considered in the atrophy analyses. Regional atrophy differences between DLB and controls did not survive false discovery rate (FDR) correction for multiple comparisons, although increased [^18^F]flortaucipir SUVR (standard uptake value ratio) was associated with increased middle temporal pole atrophy [25].

The remaining structural MRI-studies evaluated atrophy and ventricular expansion, specifically accounting for AD-copathology. The findings from these studies also show that progressive atrophy is related to amyloid pathology, with more progression when there is AD-copathology. Two studies with autopsy-confirmed diagnoses reported that patients with AD and mixed DLB + AD exhibited steeper whole-brain atrophy rates and ventricular expansion compared to “pure” DLB and controls, with regional differences most notable in the temporoparietal cortex, hippocampus, and amygdala. Pure DLB and controls were comparable, as were mixed DLB + AD and AD [26], [27]. In one of these studies, the differences between AD, DLB and mixed DLB + AD were limited, aside from a trend toward greater ventricular expansion in mixed DLB + AD compared to DLB [26]. Supporting these findings, Sarro et al. [28], showed in 22 DLB patients that a higher burden of amyloid pathology at baseline, assessed with [^11^C]PiB amyloid-PET, was associated with faster neurodegeneration in several (sub)cortical regions as well as steeper rates of ventricular expansion.

**Fig. 3:**
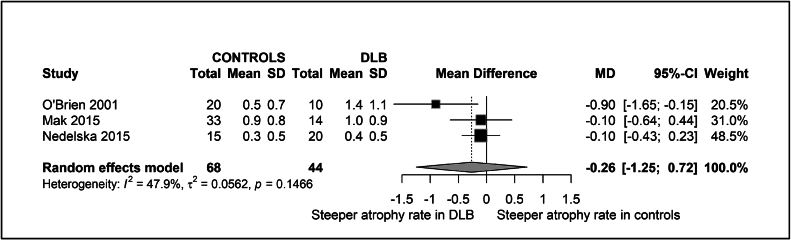
Forest plot of comparison for annualized percentage change in whole brain atrophy: DLB vs controls.

**Fig. 4:**
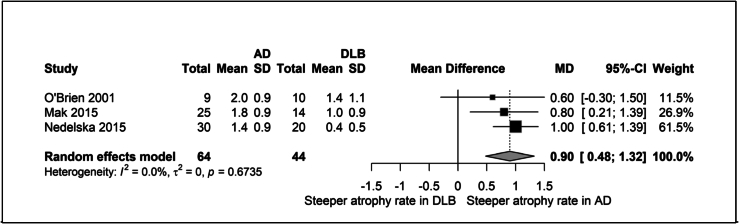
Forest plot of comparison for annualized percentage change in whole brain atrophy: DLB vs AD.

We performed meta-analysis on three of the aforementioned studies [21], [22], [27] that assessed annualized whole-brain atrophy rates in a combined number of 44 DLB, 64 CE and 68 controls over a period of 1–2 years. The results of this meta-analysis confirmed that patients with DLB do not differ significantly from controls (MD -0.26 [−1.25;0.72], *p* = 0.37], but they exhibit lower atrophy rates compared to AD patients (MD 0.90 [0.48;1.32], *p* = 0.01). The forest plot of the meta-analysis for annualized percentage change in whole brain atrophy in DLB versus controls is shown in Fig. 3, and DLB versus AD in Fig. 4. To account for methodological heterogeneity across these studies, a fixed-effects model was applied as a sensitivity analysis. This did not change the results (data not shown).

Five of the aforementioned studies assessed associations between atrophy rates and clinical changes. The findings are partly overlapping, but also show inconsistencies. Particularly when using broader measures, such as whole brain volume, or when combining dementia groups to increase statistical power, associations were lost: Mak et al. [23] found that in DLB, cortical thinning in the left frontal lobe was associated with cognitive decline, while motor decline correlated with right superior parietal thinning. However, a separate study, performed by Mak et al. [22] with roughly the same sample, found no significant associations between whole brain atrophy and cognitive decline when analyzing AD and DLB patients together. Similarly, Sarro et al. [28] reported no link between atrophy in 10 ROIs and MMSE changes, but did find that ventricular expansion was associated with change in CDR-SoB scores. Nedelska et al. [27] observed that whole brain, hippocampal, and amygdalar atrophy correlated with both cognitive and motor decline, but not with ventricular expansion. In MCI-LB, Kantarci et al. [24] found that only fusiform cortex atrophy was linked to changes in CDR-SoB scores.

#### 3.3.2 Diffusion MRI

Two studies used diffusion-weighted MRI to assess microstructural integrity. There was methodological heterogeneity among these studies regarding study duration, modeling technique, data processing, analytic methods and primary outcomes measures. Neither study assessed comorbid amyloid pathology. Firbank et al. [29] examined longitudinal changes in mean diffusivity (MD) and fractional anisotropy (FA) in DLB (*n* = 14) versus AD (*n* = 23) and controls (*n* = 32). In the DLB group, there were no regions with significant change over time in MD and FA, and changes in MD and FA were not associated with whole brain atrophy. In AD some regions had increases in MD in comparison to DLB and controls. There were no associations between longitudinal changes in MD or FA and clinical measures in the DLB group, consistent with the lack of significant changes in these imaging markers over time.

Chiu et al. [18] investigated free water (FW) and found an increase in 12 ROIs in MCI-LB/DLB patients (*n* = 39) after one and two year follow-up and in an additional 18 ROIs at two year follow-up only. In the control group (*n* = 20), no significant changes in FW over time were seen in any of the ROIs. Longitudinal increases in FW within specific ROIs were associated with motor decline after 12 months, and both motor and cognitive decline after 24 months.

### 3.4 Imaging biomarkers: SPECT

#### 3.4.1 Dopamine transporter SPECT

Two studies assessed longitudinal nigrostriatal degeneration in DLB using dopamine transporter (DAT) imaging by [^123^I]FP-CIT-SPECT. Colloby et al. [30] investigated longitudinal measures of striatal DAT binding in 20 DLB patients, 20 Parkinson's disease patients (PD), 15 Parkinson's disease dementia patients (PDD) and 22 controls. A significant reduction of the specific-to-non-displaceable binding ratio (BR_s-nd_) in both the anterior and posterior putamen was found at ±1 year follow-up in DLB. A similar trend was found for the caudate nucleus. The rate of decline was significantly higher than in controls in the caudate nucleus and posterior putamen, with a trend for the anterior putamen. Rates of decline were comparable for all striatal regions in DLB versus PD(D). A negative correlation was found between reduced posterior putaminal uptake and the rate of cognitive decline.

A more recent study also analyzed changes over time in striatal DAT binding in 11 possible MCI-LB, 25 probable MCI-LB, 20 MCI-AD and 29 controls. Durcan et al. [16] found significant decline in putaminal specific binding ratio (SBR) in both possible and probable MCI-LB. This was not found in controls and MCI-AD. In both MCI-LB groups the median annual decline in SBR was greater in the putamen than in the caudate nucleus, with no significant difference between MCI-LB groups. There were significant but weak correlations between longitudinal striatal SBR and cognition and motor functioning (*r* = 0.15, *r* = −0.14). These findings suggest that putaminal dopaminergic degeneration follows a similar course across early disease phenotypes.

#### 3.4.2 Cerebral perfusion SPECT

One study with repeated measures of [^99m^Tc]HMPAO-SPECT investigated cerebral perfusion in DLB (*n* = 18) and PDD (*n* = 17) compared to controls (*n* = 34) [31]. Increased left putaminal perfusion was found in DLB versus controls. Increased perfusion in the right caudate nucleus correlated with motor decline. It is hypothesized that the left putaminal increase reflects a compensatory mechanism in a relatively early stage of dopaminergic degeneration, as it was not found in PDD where putaminal degeneration is more advanced.

### 3.5 Imaging biomarkers: PET

#### 3.5.1 FDG-PET

One study investigated repeated [^18^F]FDG-PET in MCI-LB (*n* = 37), probable DLB (*n* = 35) and controls (*n* = 100), to assess cerebral glucose metabolism changes over 3.8 ± 2.3 years. Several ROIs in MCI-LB and DLB showed steeper SUVR decline than controls. A combined meta-ROI showed similar but weaker effects. In the total Lewy body group, faster FDG-SUVR decline in the separate ROIs and meta-ROI was associated with faster increase in a disease-severity score. However, after FDR-correction no significant decline was found in ‘high likelihood DLB’ patients in a neuropathologic subcohort (n = 10), whereas patients with ‘Lewy body disease with AD-copathology’ (*n* = 8) had steeper decline across most ROIs and the meta-ROI [19].

#### 3.5.2 Amyloid-PET

One study used repeated [^11^C]PiB amyloid-PET to track cerebral amyloid-β deposition in 35 DLB patients over ±1 year. Deposition rates did not differ from 140 controls, despite higher baseline SUVRs in DLB, particularly in APOE ε4 carriers. SUVR trajectories followed an inverted U-shape in both groups, with initial acceleration, a peak around 1.8, and subsequent deceleration. In DLB, greater longitudinal SUVR changes were associated with increased disease severity and cognitive decline. This supports aforementioned results showing more disease progression in DLB with AD-copathology [32].

#### 3.5.3 Tau-PET

Repeated tau-PET in 22 DLB patients showed increased [^18^F]flortaucipir uptake in inferior parietal, middle and superior occipital cortices, and the fusiform gyrus compared to 22 controls, although differences did not survive FDR-correction. Tau accumulation did not differ between amyloid-positive and -negative DLB. Increased SUVR was associated with longitudinal atrophy in the middle temporal cortex. Also, increased SUVR in the fusiform gyrus and occipital cortices correlated with increased disease severity and cognitive decline, even when regions were combined into a meta-ROI [25].

### 3.6 Fluid biomarkers: Plasma p-tau181

One study investigated plasma p-tau181 in 37 MCI patients (AD and DLB) over 1–2 years. This longitudinal analysis was limited to a subcohort of the full study population with little information on composition or demographics [20]. The authors reported a non-significant 3% annual increase, without group differences or associations with cognition.

### 3.7 Fluid biomarkers: CSF Aβ_42_, Aβ_40_, p-tau181, t-tau

One study assessed longitudinal CSF AD biomarkers, also in a subcohort of the full study population: 27 DLB, 5 CE, 2 MCI-AD and 29 controls [17]. In DLB, no differences were observed after 12 months for Aβ_42/40_-ratio, t-tau, or p-tau181, though Aβ_42_ and Aβ_40_ increased individually without altering the proportion of amyloid-positive patients. No cognitive or motor changes were found. In controls, significant changes were found in Aβ_42_, Aβ_42/40_-ratio, and p-tau181 but not Aβ_40_ or t-tau with follow-up duration varying widely between DLB and controls (12 versus 6–60 months, respectively). By amyloid/tau/neurodegeneration profile, DLB patients with A + T- showed increases in Aβ_42/40_-ratio, Aβ_40_ and Aβ_42_, while DLB patients with A + T+ showed increases in Aβ_40_ and Aβ_42_ only. Interestingly, some A + T- patients normalized to A-T- over time, suggesting a potential subtype warranting further investigation since this was not found for patients with A + T+ at baseline.

## 4 Discussion

In this systematic review of 17 studies, FDG-PET and DAT-SPECT emerged as the strongest progression modalities, based on their ability to detect longitudinal biomarker changes correlating with clinical progression. However, current evidence remains insufficient to establish either as a validated monitoring biomarker.

The FDG-PET study reported longitudinal changes in brain hypometabolism that correlated with increasing clinical disease severity [19]. The identification of region-specific patterns of hypometabolism corresponding to different disease stages, as well as neuropathological confirmation for a subcohort added robustness to these findings. Because this evidence is derived from a single study, further research is needed to confirm and expand upon this.

Two DAT-SPECT studies also showed changes in both biomarker and clinical outcomes [16], [30], suggesting putaminal dopaminergic degeneration as a pivotal phenomenon in DLB, correlating with cognitive and motor decline. Results from the cerebral perfusion study supported this. Despite small sample sizes (*n* = 20–36) limiting statistical power, these findings suggest that assessing (putaminal) dopaminergic degeneration alongside clinical performance has potential as a progression measure. Emerging imaging methods offer advantages over DAT-SPECT. DAT-PET is suggested to provide better quantitative accuracy, resolution, sensitivity and multimodal target assessment [33], but its cost and limited clinical availability could restrict widespread use of nuclear imaging. In this context, neuromelanin MRI may represent a more accessible and feasible alternative for assessing dopaminergic degeneration [34], [35].

Additionally, Chiu et al. [18] reported changes in both biomarker and clinical measures in (MCI-)DLB in their diffusion MRI study. The small sample size and lack of adjustment for potential AD-copathology limit the evidence supporting diffusion MRI as a progression measure in DLB.

The majority of studies included in this review repeated structural MRI measurements. Comparisons between DLB and controls, and between DLB and AD suggest that progressive brain atrophy is largely driven by amyloid-pathology. This was supported by results from meta-analysis and emphasized by studies that assessed amyloid-copathology and underscored differences between DLB and DLB + AD. This interpretation aligns with autopsy studies, indicating that neuritic amyloid plaques are likely drivers of neuronal loss, whereas α-synuclein and diffuse amyloid plaques (commonly seen in DLB but not considered diagnostic for AD-pathology at autopsy) do not significantly contribute to neurodegeneration [23], [25].

In the amyloid- and tau-PET studies, the rate of protein deposition in DLB was either comparable to controls or changes did not survive FDR-correction [25], [32]. The amyloid-PET study reported similar patterns of change in SUVR across DLB, controls and AD, suggesting that amyloid accumulation follows typical progression even when present in non-AD diagnoses. Although longitudinal changes in tau SUVR did not survive FDR-correction, the authors reported that regional tau increases were associated with clinical deterioration. This association was not replicated in the plasma p-tau181 study [20]. Of note, as AD-biomarkers primarily reflect AD-copathology in DLB, they are not suitable as stand-alone progression measures for DLB itself.

Mapping the biomarkers onto the BEST framework [6], FDG-PET and DAT-SPECT emerged as the strongest candidate monitoring biomarkers. HMPAO-SPECT and diffusion MRI showed preliminary monitoring potential, though evidence remains limited. Structural MRI, amyloid-PET, tau-PET, plasma and CSF AD-biomarkers showed more potential for diagnostic or prognostic use, as their longitudinal trajectories are largely driven by AD-copathology (Table 2). In clinical trials, AD-specific biomarkers would therefore be best suited for stratifying participants based on AD-copathology status, and may gain relevance with the emergence of anti-amyloid treatments. DAT-SPECT is the only modality that would be usable for participant selection and as a surrogate endpoint. Durcan et al. [16] projected ±6 years from normal baseline uptake to abnormal putaminal binding in MCI-LB, favouring patient selection for trials with pre-existing dopaminergic deficits. Sample size estimates from the FDG-PET study suggest that FDG-PET outperforms clinical outcomes as a trial endpoint, supporting its potential as a secondary endpoint in phase-2 trials [19]. Given the slow rate of change in pure DLB, using structural MRI as a secondary endpoint would require impractically long trial durations. It may therefore serve better as a safety biomarker. Trial roles are summarized in Table 2, alongside modality-specific strengths and limitations.

**Table 2:**
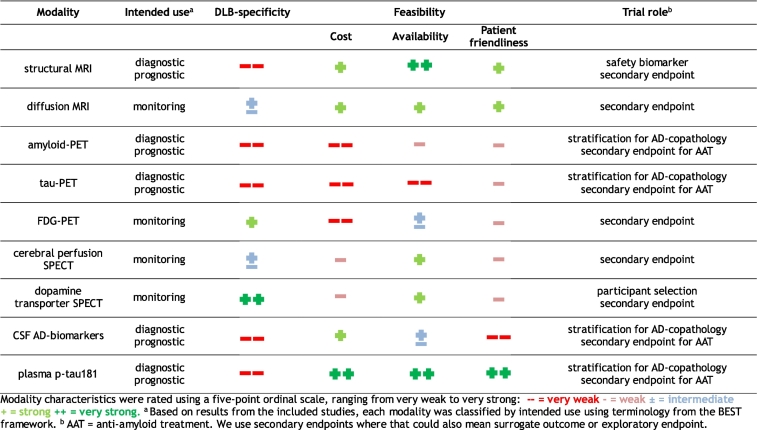
Strengths and limitations of biomarkers in included studies.

Our findings align with the previous selective review of Zarkali et al. (2025) [10]. The present systematic review directly addresses the lack of longitudinal data they identified as a critical gap, and extends their work by mapping findings onto the BEST framework and translating evidence into trial-relevant context (Table 2).

A critical gap in the current toolkit for DLB-specific trial design is the complete absence of longitudinal studies assessing α-synuclein-specific biomarkers. Ideally, an α-synuclein-specific monitoring biomarker is characterized by DLB-specificity, quantitative output with sufficient dynamic range to detect longitudinal change, absence of ceiling effects in early disease stages, temporal correlation with clinical decline and feasibility in terms of cost, availability and patient burden. The most promising diagnostic biomarker for α-synucleinopathy is the CSF seeding amplification assay (SAA) [36], but its binary output currently limits longitudinal monitoring. While evidence remains preliminary, emerging work suggests that kinetic SAA-parameters [37], oligomeric α-synucleinopathy assays [38] and α-synuclein-PET imaging [39] could provide quantification of pathology burden rather than binary results. Additionally, growing evidence supports that CSF DOPA decarboxylase, being a quantitative measure of dopaminergic degeneration, has potential as a DLB-specific progression measure [40].

A key limitation in this review is the substantial heterogeneity across studies. Differences in imaging protocols, diagnostic criteria, control definitions, and cohort composition, including enrichment for MCI-cases, presence of parkinsonism, and degree of AD-copathology, limit the generalizability of progression estimates, precluding formal meta-analyses beyond structural MRI. In addition, average follow-up duration was relatively short (±18 months), limiting the informativeness of annualized rates of change and potentially resulting in insufficient time to detect significant longitudinal changes. In two studies, samples consisted of longitudinal subcohorts within originally cross-sectional study designs, limiting generalizability and quantitative inference. Another important consideration is that clinical progression was assessed only with (global) cognitive and motor outcomes, under-leveraging DLB's phenotypic richness and therefore providing a rather narrow operationalization of ‘clinical progression’. Finally, differences in individual biomarker- and clinical trajectories may be attributable to distinct differences in α-synucleinopathy onset and spread [41], a phenomenon increasingly recognized in emerging staging systems [42], [43] that warrants further investigation.

Recommendations for future directions include enriching study samples across the full clinical disease spectrum to enable stage-specific biomarker evaluation, and mapping biomarker dynamics to pre-specified composite clinical outcomes that are tailored to all DLB-specific core features and relevant cognitive domains such as attention, executive functioning and visuoperceptual abilities. Also, longitudinal studies are needed that include all current DLB-specific indicative biomarkers. Addressing the absence of validated DLB-specific monitoring biomarkers, including both α-synuclein-specific measures and quantitative measures of dopaminergic degeneration, should be considered a priority for the field.

To conclude, due to the scarcity of and heterogeneity across studies, methodological limitations, and the limited specificity and insufficient longitudinal discriminative ability of the biomarkers, based on this review there is no validated biomarker to demonstrate disease progression in DLB; the field therefore currently lacks a monitoring biomarker.

## CRediT authorship contribution statement

**Juliette L. van Alphen:** Writing – review & editing, Writing – original draft, Visualization, Validation, Software, Resources, Project administration, Methodology, Investigation, Formal analysis, Data curation, Conceptualization. **Federico E. Pozzi:** Writing – review & editing, Writing – original draft, Software, Investigation, Data curation. **Jan Booij:** Writing – review & editing. **Frederik Barkhof:** Writing – review & editing. **Mara ten Kate:** Writing – review & editing. **Charlotte E. Teunissen:** Writing – review & editing. **Wiesje M. van der Flier:** Writing – review & editing, Supervision, Methodology. **Afina W. Lemstra:** Writing – review & editing, Supervision, Methodology, Data curation, Conceptualization.

## Declaration of competing interest

The authors declare the following financial interests/personal relationships which may be considered as potential competing interests:Juliette van Alphen reports financial support was provided by Gieskes-Strijbis Fund Foundation. Juliette van Alphen reports financial support was provided by TAP-Dementia funded by Netherlands Organisation for Health Research and Development. Jan Booij reports a relationship with GE Healthcare that includes: consulting or advisory. Frederik Barkhof reports a relationship with Biogen that includes: board membership. Frederik Barkhof reports a relationship with Merck that includes: board membership. Frederik Barkhof reports a relationship with Eisai that includes: board membership. Frederik Barkhof reports a relationship with Prothena that includes: board membership. Frederik Barkhof reports a relationship with Combinostics that includes: board membership. Frederik Barkhof reports a relationship with Scottish Brain Sciences that includes: board membership. Frederik Barkhof reports a relationship with IXICO that includes: board membership. Frederik Barkhof reports a relationship with Alzheimer Europe that includes: board membership. Frederik Barkhof reports a relationship with Roche that includes: consulting or advisory. Frederik Barkhof reports a relationship with Celltrion that includes: consulting or advisory. Frederik Barkhof reports a relationship with Merck that includes: consulting or advisory. Frederik Barkhof reports a relationship with Bracco that includes: consulting or advisory. Charlotte E. Teunissen reports a relationship with European Commission Marie Curie International Training Network grant agreement No 860197 (MIRIADE) and No 101119596 (TAME) that includes: funding grants. Charlotte E. Teunissen reports a relationship with Innovative Medicines Initiative 3TR (Horizon 2020, grant no 831434) that includes: funding grants. Charlotte E. Teunissen reports a relationship with EPND (IMI 2 Joint Undertaking (JU), grant No. 101034344) that includes: funding grants. Charlotte E. Teunissen reports a relationship with JPND (bPRIDE, CCAD) that includes: funding grants. Charlotte E. Teunissen reports a relationship with European Partnership on Metrology, cofinanced from the European Union's Horizon Europe Research and Innovation Programme and by the Participating States 22HLT07 NEuroBioStand that includes: funding grants. Charlotte E. Teunissen reports a relationship with Horizon Europe (PREDICTFTD, 101156175) that includes: funding grants. Charlotte E. Teunissen reports a relationship with CANTATE project funded by Alzheimer's Drug Discovery Foundation that includes: funding grants. Charlotte E. Teunissen reports a relationship with Alzheimer's Association that includes: funding grants. Charlotte E. Teunissen reports a relationship with The Michael J Fox Foundation that includes: funding grants. Charlotte E. Teunissen reports a relationship with Health Holland that includes: funding grants. Charlotte E. Teunissen reports a relationship with Dutch Research Council that includes: funding grants. Charlotte E. Teunissen reports a relationship with The Selfridges Group Foundation that includes: funding grants. Charlotte E. Teunissen reports a relationship with Alzheimer Netherlands that includes: funding grants. Charlotte E. Teunissen reports a relationship with Topsector Life Sciences & Health that includes: funding grants. Charlotte E. Teunissen reports a relationship with Aribio that includes: consulting or advisory and speaking and lecture fees. Charlotte E. Teunissen reports a relationship with Biogen that includes: consulting or advisory and speaking and lecture fees. Charlotte E. Teunissen reports a relationship with Beckman-Coulter that includes: consulting or advisory and speaking and lecture fees. Charlotte E. Teunissen reports a relationship with Cognition Therapeutics that includes: consulting or advisory and speaking and lecture fees. Charlotte E. Teunissen reports a relationship with Danaher that includes: consulting or advisory and speaking and lecture fees. Charlotte E. Teunissen reports a relationship with Eisai that includes: consulting or advisory and speaking and lecture fees. Charlotte E. Teunissen reports a relationship with Eli Lilly that includes: consulting or advisory and speaking and lecture fees. Charlotte E. Teunissen reports a relationship with Janssen that includes: consulting or advisory and speaking and lecture fees. Charlotte E. Teunissen reports a relationship with Merck that includes: consulting or advisory and speaking and lecture fees. Charlotte E. Teunissen reports a relationship with Neurogen Biomarking that includes: consulting or advisory and speaking and lecture fees. Charlotte E. Teunissen reports a relationship with Nordic Biosciences that includes: consulting or advisory and speaking and lecture fees. Charlotte E. Teunissen reports a relationship with Novo Nordisk that includes: consulting or advisory and speaking and lecture fees. Charlotte E. Teunissen reports a relationship with Novartis that includes: consulting or advisory and speaking and lecture fees. Charlotte E. Teunissen reports a relationship with Olink that includes: consulting or advisory and speaking and lecture fees. Charlotte E. Teunissen reports a relationship with Quanterix that includes: consulting or advisory and speaking and lecture fees. Charlotte E. Teunissen reports a relationship with Roche that includes: consulting or advisory and speaking and lecture fees. Charlotte E. Teunissen reports a relationship with Sanofi that includes: consulting or advisory and speaking and lecture fees. Charlotte E. Teunissen reports a relationship with Veravas that includes: consulting or advisory and speaking and lecture fees. Wiesje M. van der Flier reports a relationship with Netherlands Organisation for Health Research and Development that includes: funding grants. Wiesje M. van der Flier reports a relationship with NWO that includes: funding grants. Wiesje M. van der Flier reports a relationship with EU-JPND that includes: funding grants. Wiesje M. van der Flier reports a relationship with EU-IHI that includes: funding grants. Wiesje M. van der Flier reports a relationship with Alzheimer Netherlands that includes: funding grants. Wiesje M. van der Flier reports a relationship with Hersenstichting that includes: funding grants. Wiesje M. van der Flier reports a relationship with CardioVasculair Onderzoek Nederland that includes: funding grants. Wiesje M. van der Flier reports a relationship with Health Holland that includes: funding grants. Wiesje M. van der Flier reports a relationship with Topsector Life Sciences & Health that includes: funding grants. Charlotte E. Teunissen reports a relationship with Alzheimer's Drug Discovery Foundation that includes: funding grants. Wiesje M. van der Flier reports a relationship with Dioraphte Foundation that includes: funding grants. Wiesje M. van der Flier reports a relationship with Noaber Foundation that includes: funding grants. Wiesje M. van der Flier reports a relationship with Pieter Houbolt Fonds that includes: funding grants. Wiesje M. van der Flier reports a relationship with Gieskes-Strijbis Fund Foundation that includes: funding grants. Wiesje M. van der Flier reports a relationship with Stichting Equilibrio that includes: funding grants. Wiesje M. van der Flier reports a relationship with Edwin Bouw Fonds that includes: funding grants. Wiesje M. van der Flier reports a relationship with Pasman Stichting that includes: funding grants. Wiesje M. van der Flier reports a relationship with Philips that includes: funding grants. Wiesje M. van der Flier reports a relationship with Biogen MA Inc. that includes: funding grants. Wiesje M. van der Flier reports a relationship with Novartis-NL that includes: funding grants. Wiesje M. van der Flier reports a relationship with LIFE-MI that includes: funding grants. Wiesje M. van der Flier reports a relationship with AVID that includes: funding grants. Wiesje M. van der Flier reports a relationship with Roche BV that includes: funding grants. Wiesje M. van der Flier reports a relationship with Eli-Lilly-NL that includes: funding grants. Wiesje M. van der Flier reports a relationship with Fujifilm that includes: funding grants. Wiesje M. van der Flier reports a relationship with Eisai that includes: funding grants. Wiesje M. van der Flier reports a relationship with Combinostics that includes: funding grants. Co-author has research agreements with ADDI, Merck, Biogen, GE Healthcare, Icometrix, Roche - FB. Co-author is co-founder and shareholder of Queen Square Analytics LTD - FB. Co-author is recipient of ABOARD, which is a public-private partnership receiving funding from ZonMW (#73305095007) and Health Holland - CET. Co-author is recipient of TAP-dementia, a ZonMw funded project (#10510032120003) in the context of the Dutch National Dementia Strategy - CET. Co-author has research contracts with Acumen, ADx Neurosciences, AC-Immune, Alamar, Aribio, Axon Neurosciences, Beckman-Coulter, BioConnect, Bioorchestra, Brainstorm Therapeutics, C2N diagnostics, Celgene, Cognition Therapeutics, EIP Pharma, Eisai, Eli Lilly, Fujirebio, Instant Nano Biosensors, Merck, Muna, Nitrase Therapeutics, Novo Nordisk, Olink, PeopleBio, Quanterix, Roche, Sysmex, Toyama, Vaccinex, Vivoryon - CET. Co-author is is editor in chief of Alzheimer Research and Therapy - CET. Co-author serves on editorial boards of Molecular Neurodegeneration, Alzheimer's & Dementia, Neurology: Neuroimmunology & Neuroinflammation, Medidact Neurologie/Springer - CET. Co-author is committee member to define guidelines for Cognitive disturbances, and one for acute Neurology in the Netherlands - CET. Co-author is recipient of ABOARD, which is a public-private partnership receiving funding from ZonMW (#73305095007) and Health Holland - WF. Co-author is recipient of TAP-dementia (www.tap-dementia.nl), receiving funding from ZonMw (#10510032120003). TAP-dementia receives co-financing from Avid Radiopharmaceuticals, Roche, and Amprion - WF. Co-author is recipient of IHI- PROMINENT (#101112145) and IHI-AD-RIDDLE (#101132933). PROMINENT and AD-RIDDLE are supported by the Innovative Health Initiative Joint Undertaking (IHI JU). The JU receives support from the European Union's Horizon Europe research and innovation programme and COCIR, EFPIA, EuropaBio, MedTech Europe and Vaccines Europe, with Davos Alzheimer's Collaborative, Combinostics OY., Cambridge Cognition Ltd., C2N Diagnostics LLC, and neotiv GmbH. All funding is paid to her institution - WF. C0-author holds the Pasman chair - WF. Co-author has been an invited speaker at Biogen MA Inc., Danone, Eisai, WebMD Neurology (Medscape), NovoNordisk, Springer Healthcare, European Brain Council. All funding is paid to her institution - WF. Co-author is is consultant to Oxford Health Policy Forum CIC, Roche, Biogen MA Inc., Eisai, Eli-Lilly, Owkin France, Nationale Nederlanden Ventures. All funding is paid to her institution - WF. Co-author participated in advisory boards of Biogen MA Inc., Roche, and Eli Lilly - WF. Co-author is member of the steering committee of phase 3 EVOKE/EVOKE+ studies (NovoNordisk). Co-author is member of the steering committee op phase 3 Trontinemab study (Roche). All funding is paid to her institution - WF. Co-author is member of the steering committee of PAVE, and Think Brain Health. WF is chair of the Scientific Leadership Group of InRAD - WF. Co-author was associate editor of Alzheimer, Research & Therapy in 2020/2021 - WF. Co-author is associate editor at Brain - WF. Co-author is member of Supervisory Board (Raad van Toezicht) Trimbos Instituut - WF. If there are other authors, they declare that they have no known competing financial interests or personal relationships that could have appeared to influence the work reported in this paper.
