# Supplementary material for: Longitudinal biomarkers in dementia with Lewy bodies: A systematic review and meta-analysis
Source: Clin Park Relat Disord. 2026 Jun 17;15:100470. doi: 10.1016/j.prdoa.2026.100470 (PMC13316175; doi:10.1016/j.prdoa.2026.100470)
Supplement: Supplementary file 1 — Supplementary material 1 [file mmc1.docx]

**PubMed**

("Longitudinal Studies"[Mesh:NoExp] OR repe* OR trajector*) AND ( "Amyloid beta-Peptides/blood"[Mesh] OR "Amyloid beta-Peptides/cerebrospinal fluid"[Mesh] OR “blood” [Subheading] OR "cerebrospinal fluid" [Subheading] OR "tau Proteins/blood"[Mesh] OR "tau Proteins/cerebrospinal fluid"[Mesh] OR "Neurofilament Proteins/blood"[Mesh] OR "Neurofilament Proteins/cerebrospinal fluid"[Mesh] OR "Magnetic Resonance Imaging"[Mesh] OR "Positron-Emission Tomography"[Mesh] OR "Biomarkers/analysis"[Mesh] OR "Biomarkers/blood"[Mesh] OR "Biomarkers/cerebrospinal fluid"[Mesh] OR "alpha-Synuclein/analysis"[Mesh] OR "alpha-Synuclein/blood"[Mesh] OR "alpha-Synuclein/cerebrospinal fluid"[Mesh]) AND ("Lewy Body Disease"[Mesh] OR "Parkinson Disease"[Mesh])

**Embase**

('longitudinal'/mj OR repe* OR trajector*) AND (biomarker* OR 'blood'/mj OR 'csf'/mj OR 'pet'/mj OR 'mri'/mj OR 'amyloid'/mj OR 'tau'/mj OR 'neurofilament'/mj OR 'alpha synuclein'/mj) AND ('dementia lewy bod*' OR parkinson OR 'lewy body disease'/mj) AND [humans]/lim

Mapping: "map to preferred term in Emtree" and "limit to terms indexed in articles as major focus"

**Scopus**

TITLE-ABS-KEY ( ( longitudinal OR repe* OR trajector* ) AND ( biomarker OR pet OR mri OR amyloid OR tau OR neurofilament OR alpha-synuclein ) AND ( "Dementia Lewy Bod*" OR parkinson ) ) AND SUBJAREA ( medi )

**Web of Science**

AB=((longitudinal OR repe* OR trajector*) AND (biomarker* OR blood OR CSF OR PET OR MRI OR amyloid OR tau OR neurofilament OR alpha-synuclein) AND (“Dementia Lewy Bod*” OR Parkinson OR “Lewy Body disease”)) OR TI=((longitudinal OR repe* OR trajector*) AND (biomarker* OR blood OR CSF OR PET OR MRI OR amyloid OR tau OR neurofilament OR alpha-synuclein) AND (“Dementia Lewy Bod*” OR Parkinson OR “Lewy Body disease”)) OR AK=((longitudinal OR repe* OR trajector*) AND (biomarker* OR blood OR CSF OR PET OR MRI OR amyloid OR tau OR neurofilament OR alpha-synuclein) AND (“Dementia Lewy Bod*” OR Parkinson OR “Lewy Body disease”))

**Cochrane Central Register of Controlled Trials (CENTRAL)**

([mh ^“longitudinal study”] OR repe* OR trajectory*) AND ([mh "Amyloid beta-Peptides"/BL,CF] OR [mh "tau Proteins"/BL,CF] OR [mh "Neurofilament Proteins"/BL,CF] OR [mh "Magnetic Resonance Imaging"] OR [mh "Positron-Emission Tomography"] OR [mh "Biomarkers"/BL,CF] OR [mh "alpha-Synuclein"/BL,CF] OR [mh /CF,BL]) AND ([mh “Lewy body disease”] OR [mh “Parkinson Disease”])ß
