# Supplementary material for: Longitudinal biomarkers in dementia with Lewy bodies: A systematic review and meta-analysis
Source: Clin Park Relat Disord. 2026 Jun 17;15:100470. doi: 10.1016/j.prdoa.2026.100470 (PMC13316175; doi:10.1016/j.prdoa.2026.100470)
Supplement: Supplementary file 2 — Supplementary material 2 [file mmc2.docx]

**Critical appraisal checklist for cohort studies, developed by the Johanna Brigg Institute**

|  | **Question** | | | | | | | | | | | **Quality** | |
| --- | --- | --- | --- | --- | --- | --- | --- | --- | --- | --- | --- | --- | --- |
|  | 1 | 2 | 3 | 4 | 5 | 6 | 7 | 8 | 9 | 10 | 11 | Overall score | Appraisal |
| **MRI-studies** | | | | | | | | | | | | | |
| O’Brien 2001 | yes | yes | yes | yes | yes | NA | yes | yes | yes | NA | yes | 9 | high |
| Whitwell 2007 | yes | yes | yes | yes | yes | NA | yes | UC | yes | NA | yes | 8 | intermediate |
| Mak 2015^a^ | yes | yes | yes | yes | yes | NA | yes | yes | no | yes | yes | 9 | high |
| Mak 2015^b^ | yes | yes | yes | yes | yes | NA | yes | yes | no | yes | yes | 9 | high |
| Nedelska 2015 | yes | yes | yes | yes | yes | NA | yes | yes | yes | NA | yes | 9 | high |
| Sarro 2016 | NA | NA | yes | yes | yes | NA | yes | yes | yes | NA | yes | 7 | intermediate |
| Kantarci 2022 | yes | yes | yes | yes | yes | NA | yes | yes | yes | NA | yes | 9 | high |
| Firbank 2016 | yes | yes | yes | yes | yes | NA | yes | yes | no | yes | yes | 9 | high |
| Chiu 2024 | yes | yes | yes | yes | yes | NA | yes | yes | UC | UC | yes | 8 | intermediate |
| **PET-studies** | | | | | | | | | | | | | |
| Nedelska 2019 | yes | yes | yes | yes | yes | NA | yes | yes | yes | NA | yes | 9 | high |
| Chen 2022 | yes | yes | yes | yes | yes | NA | yes | UC | yes | NA | yes | 8 | intermediate |
| Ferreira 2025 | yes | yes | yes | yes | yes | NA | yes | yes | UC | UC | yes | 8 | intermediate |
| **SPECT-studies** | | | | | | | | | | | | | |
| Colloby 2005 | yes | yes | yes | yes | yes | NA | yes | yes | no | no | yes | 8 | intermediate |
| Firbank 2005 | yes | yes | yes | yes | yes | NA | yes | yes | no | no | yes | 8 | intermediate |
| Durcan 2023 | yes | yes | yes | yes | yes | NA | yes | yes | yes | NA | yes | 9 | high |
| **biofluid-studies** | | | | | | | | | | | | | |
| Thomas 2022 | UC | yes | yes | yes | yes | NA | yes | yes | UC | UC | yes | 7 | intermediate |
| Abdelnour 2022 | UC | yes | yes | yes | yes | NA | yes | yes | UC | UC | yes | 7 | intermediate |
| Jain 2024 | UC | yes | yes | yes | yes | NA | yes | yes | UC | UC | yes | 7 | intermediate |

NA = not applicable, UC = unclear

*Questions of JBI Critical Appraisal Checklist for Cohort Studies 1) Were the two groups similar and recruited from the same population? 2) Were the exposures measured similarly to assign people to both exposed and unexposed groups?^*^ 3) Was the exposure measured in a valid and reliable way? 4) Were confounding factors identified? 5) Were strategies to deal with confounding factors stated? 6) Were the groups/participants free of the outcome at the start of the study (or at the moment of exposure)?^**^ 7) Were the outcomes measured in a valid and reliable way? 8) Was the follow up time reported and sufficient to be long enough for outcomes to occur? 9) Was follow up complete, and if not, were the reasons to loss to follow up described and explored? 10) Were strategies to address incomplete follow up utilized? 11) Was appropriate statistical analysis used?*

*^*^In this context, ‘exposure‘ refers to the assessment of the biomarker*

*^**^All included studies assessed outcome measures defined as change over time (e.g. longitudinal change in biomarker levels and/or clinical scores). Therefore, this criterion is not applicable to the included studies in this systematic review, as participants cannot be free of a change-based outcome at baseline.*
